# Ankle-brachial index (ABI) and quiescent-interval single shot (QISS) MRA in peripheral arterial disease (PAD): comparison of diagnostic accuracy and need for additional imaging procedures

**DOI:** 10.1186/1532-429X-13-S1-P391

**Published:** 2011-02-02

**Authors:** Emily VM Ward, Asad A Usman, Philip A Hodnett, James C Carr, Robert R Edelman

**Affiliations:** 1 Northshore University Healthsystem, Evanston, Chicago, IL, USA; 2 Northwestern Memorial Hospital, Chicago, IL, USA; 3 NYU Langone Medical Center, New York, NY, USA

## Objectives

To determine whether a rapid, non-contrast MRA technique might provide superior accuracy to ABI and reduce the need for additional imaging procedures prior to a revascularization procedure.

## Background

PAD is a major cause of morbidity and accurate diagnosis is essential for optimal patient management. ABI measurement is the initial diagnostic test of choice however, accuracy is sometimes limited, e.g. in diabetic patients and with vessel calcification. Additional imaging with CTA, MRA, or DSA is often required prior to revascularization. QISS MRA is a rapid, operator-independent non-contrast MRA technique which has been reported to evaluate PAD with accuracy comparable to that of CE-MRA (1). We compared ABI and QISS MRA with respect to the following: (a) what is the accuracy for hemodynamically significant stenoses; (b) was the detected abnormality located in the symptomatic limb; (c) after imaging with QISS MRA is there an expectation for additional imaging evaluation prior to intervention?

## Methods

Using CE-MRA as the reference standard, the sensitivity and specificity of ABI and QISS MRA for 50% or greater stenosis or occlusion was determined for 60 arterial segments (5 segments per leg). In addition, the need for additional CE-MRA prior to intervention after initial evaluation with QISS MRA was rated by an interventional radiologist.

## Results

The mean age was 67 years (59-81, 67% male). On a segment basis, the sensitivities/specificities of ABI and QISS MRA for hemodynamically significant stenoses were 76%/83% and 96%/92% respectively (p<0.05). Significantly diseased segments were concordant with CE-MRA in 35% of ABIs and 88% of QISS MRA studies. 11.6% of segments analyzed by ABI were non-diagnostic compared with 2.9% of segments by QISS MRA. The side of the detected abnormality correlated with the symptomatic limb in 78% of ABIs and 100% of QISS MRA studies. Hemodynamically significant stenoses were identified in 21.7% of segments on both CE-MRA and QISS MRA. Of those identified with QISS MRA the need for additional imaging prior to revascularization was evaluated by an interventional radiologist. In 83.4% of cases no further imaging was required.

## Conclusion

Compared with ABI, QISS MRA provides higher accuracy for clinically relevant PAD and substantially reduces the requirement for additional imaging prior to a revascularization procedure. It has the potential to be an alternative screening examination to ABI for selected patients with PAD.
